# Interrelation of Oxidative Stress and Inflammation in Neurodegenerative Disease: Role of TNF

**DOI:** 10.1155/2015/610813

**Published:** 2015-03-05

**Authors:** Roman Fischer, Olaf Maier

**Affiliations:** Institute of Cell Biology and Immunology, University of Stuttgart, Allmandring 31, 70569 Stuttgart, Germany

## Abstract

Neuroinflammation and mitochondrial dysfunction are common features of chronic neurodegenerative diseases of the central nervous system. Both conditions can lead to increased oxidative stress by excessive release of harmful reactive oxygen and nitrogen species (ROS and RNS), which further promote neuronal damage and subsequent inflammation resulting in a feed-forward loop of neurodegeneration. The cytokine tumor necrosis factor (TNF), a master regulator of the immune system, plays an important role in the propagation of inflammation due to the activation and recruitment of immune cells via its receptor TNF receptor 1 (TNFR1). Moreover, TNFR1 can directly induce oxidative stress by the activation of ROS and RNS producing enzymes. Both TNF-induced oxidative stress and inflammation interact and cooperate to promote neurodegeneration. However, TNF plays a dual role in neurodegenerative disease, since stimulation via its second receptor, TNFR2, is neuroprotective and promotes tissue regeneration. Here we review the interrelation of oxidative stress and inflammation in the two major chronic neurodegenerative diseases, Alzheimer's and Parkinson's disease, and discuss the dual role of TNF in promoting neurodegeneration and tissue regeneration via its two receptors.

## 1. Introduction

The adult human central nervous system (CNS) consists of approximately 100 billion neurons and a similar amount of glia cells, namely, astrocytes, oligodendrocytes, and microglia [1]. The CNS parenchyma is separated from the rest of the body by the blood-brain barrier (BBB), which is formed predominantly by tight junctions of the endothelial cells of the CNS vasculature. The BBB restricts and controls the entry of nutrients and cells, including peripheral immune cells, which are almost completely absent in the healthy CNS. This has led to the concept that the CNS is an immune privileged organ. However, this concept has been modified in recent years since the CNS itself is fully immune competent and quickly responds to injury or infections [2, 3]. Moreover, cells of the peripheral innate immune system, in particular macrophages, can readily cross the BBB under pathological conditions and disturbance of the BBB, for example, in spinal cord injury, ischemia, or an adaptive immune response directed against antigens of the CNS, such as in multiple sclerosis, allows migration of T and B lymphocytes into the CNS [2]. Furthermore, systemic infections and the resulting activation of the peripheral immune system can exacerbate chronic neurodegeneration [4–6].

All types of glia cells are of relevance to maintain the homeostasis of the CNS. Of particular importance are astrocytes, which are essential for the trophic support of neurons and oligodendrocytes, and microglia, the immune cells of the CNS. Under physiological conditions the so-called resting microglia, which are kept quiescent by interaction with neuronal proteins such as CX3CL1 (fractalkine) and CD200 [7], constantly monitor the environment [8]. Alterations of the CNS environment, for example, by infection or neuronal injury, result in microglia and astrocyte activation. Depending on the damage, the first response of these cells may be the production and release of neurotrophic factors or cytokines. Prolonged neuronal damage can result in the release of proinflammatory cytokines by astrocytes and microglia, leading to the recruitment of the immune system and the development of a local inflammatory reaction. Moreover, activated microglia and astrocytes can produce reactive oxygen species (ROS), an important defense mechanism against microbial infection, which can, however, contribute to neurodegeneration [9–11].

During transient injuries microglia activation is usually not detrimental to the CNS. Although the release of proinflammatory cytokines and ROS may be harmful to neurons and oligodendrocytes, microglia are essential to remove the cellular debris and secrete neurotrophic factors after resolution of the injury and are thus essential for tissue regeneration [7]. If, however, the insult persists for a long period this may result in permanent activation of microglia and thus in constant release of proinflammatory cytokines and ROS. This is particularly relevant in the context of chronic neurodegenerative diseases such as Alzheimer's disease, Parkinson's disease, and multiple sclerosis. Indeed, neuroinflammation, that is, the presence of proinflammatory cytokines and activated immune cells, is a common feature of all neurodegenerative diseases [2, 3, 12].

Here we review the relationship between oxidative stress and inflammation in chronic neurodegenerative disease. In particular we will focus on the role of the tumor necrosis factor (TNF), which is released by activated astrocytes and microglia and which can exacerbate inflammation and promote the release of ROS from microglia thereby promoting neurodegeneration. Interestingly, TNF not only elicits detrimental and degenerative responses. Particularly in the nervous tissue TNF can also ameliorate immune responses and promote regeneration and neuroprotection [13, 14].

## 2. General Aspects of Neurodegenerative Diseases

We will restrict our discussion on the interrelation of oxidative stress and inflammation in the two major chronic neurodegenerative diseases, namely, Alzheimer's disease (AD) and Parkinson's disease (PD). Where applicable we will correlate certain aspects to multiple sclerosis (MS), the predominant inflammatory disease of the CNS.

AD is characterized by memory impairment and cognitive decline due to neuronal loss mainly in the neocortex and the hippocampus. The main histopathological hallmark is the formation of extracellular plaques consisting predominantly of aggregates formed by amyloid beta (A*β*), which are generated by proteolytic processing of the C-terminus of the amyloid precursor protein (APP) by specific proteases, *β*-secretase and *γ*-secretase. Moreover, intracellular protein aggregates, the so-called neurofibrillary tangles, which consist predominantly of hyperphosphorylated and misfolded tau protein, are characteristically found in the neurons of AD patients. Since tau is important for stability of microtubules its deregulation may result in impaired axonal transport. Although more than 90% of AD cases are sporadic, there are also familial forms of this disease, which are caused by mutations in APP or presenilin, the catalytic subunit of *γ*-secretase [15].

PD is characterized by the loss of dopaminergic neurons in the substantia nigra pars compacta, which is responsible for the characteristic motor symptoms of this disease. On a subcellular level, affected neurons contain protein aggregates, the so-called Lewy bodies, which consist predominantly of *α*-synuclein. Mutations in several genes, for example, *α*-synuclein, parkin, PINK-1, and DJ-1, have been implicated in hereditary forms of PD, which comprise approximately 10% of all PD patients. Interestingly, the mutated forms of these proteins all result in dysfunction of mitochondria thus implying that oxidative stress may be instrumental in disease development and progression [16, 17].

In contrast to AD and PD, MS is characterized by an attack of virtually all cells of the immune system, especially macrophages as well as B and T lymphocytes, on oligodendrocytes, the myelinating cells of the CNS. This causes demyelination and subsequent axonal degeneration of the affected neurons. Repeated immune attacks result in the formation of plaques, which are characterized by glial scar formation, and consequently highly diverse neurological deficits [18]. MS and its animal model, experimental autoimmune encephalomyelitis (EAE), are particularly instructive to assess the role of CNS-derived cells, that is, astrocytes and microglia, versus systemic immune cells in acute inflammation of the CNS. Indeed, it has been shown that in acute EAE microglia are relatively quiescent whereas infiltrating macrophages mediate inflammation and phagocytosis [19].

In spite of the diversity of the neurodegenerative diseases, oxidative stress due to excessive production and release of ROS upon mitochondrial injury and dysfunction has been proposed as a general pathological mechanism of all major chronic neurodegenerative diseases including AD, PD, and MS (see, e.g., [9, 20–24]).

## 3. Oxidative Stress and Mitochondrial Dysfunction in Neurodegenerative Disease

**Figure 1:**
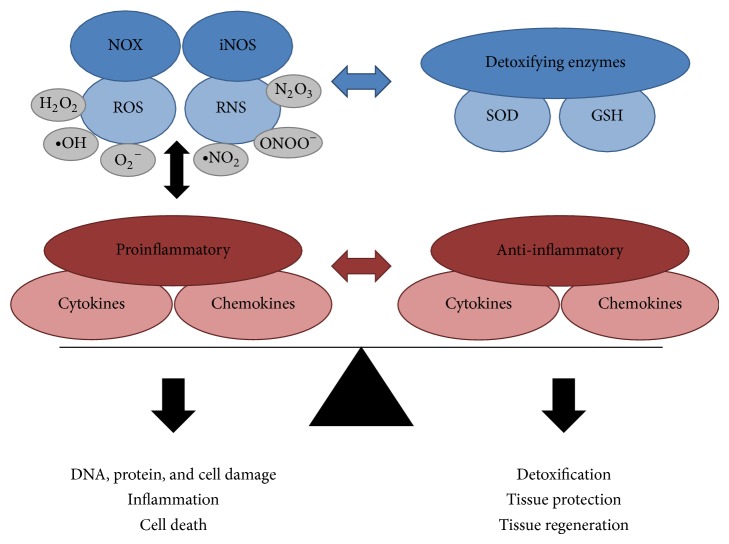
Balance between mediators of oxidative stress/inflammation and antioxidants/anti-inflammatory mediators. In a healthy organism mediators of oxidative stress and inflammation are in balance with the counteracting detoxifying and anti-inflammatory molecules. During disease this balance is shifted towards the oxidative stress and proinflammatory site, leading to DNA and protein damage, inflammation, and finally cell death.

ROS have important physiological functions, for example, by oxidation of cysteines in proteins necessary for the formation of disulfide bonds [10, 11]. Examples of ROS include the superoxide anion radical (O_2_^−∙^), hydroxyl radical (^*∙*^OH), and hydrogen peroxide (H_2_O_2_). H_2_O_2_, in particular, has been implicated in signaling pathways important for cell growth, proliferation, and survival [11, 25]. However, excessive ROS production can cause oxidative stress, which is defined as disequilibrium between ROS production and the ability to detoxify the reactive oxygen intermediates. Since oxidative stress can induce cell damage and promote inflammation [25], cells have a battery of antioxidizing molecules and enzymes to prevent the accumulation of ROS [26] (Figure 1).

During mitochondrial activity superoxide is produced in the electron transport chain (ETC). Since superoxide can inactivate proteins containing iron-sulfur clusters in the mitochondrion, it is immediately converted to H_2_O_2_ by superoxide dismutase 2 (SOD2) located in the mitochondrial matrix or SOD1 located in the cytosol [27, 28]. H_2_O_2_ can act as an oxidant and, moreover, in the presence of reduced metal ions such as ferrous iron (Fe^2+^), can be converted by the Fenton reaction into the highly reactive hydroxyl radical, the most harmful species of all ROS [17, 24]. Therefore, H_2_O_2_ is rapidly converted to water by mitochondrial glutathione (GSH) with the participation of GSH reductase and peroxiredoxins [29]. The GSH redox cycle is also important to reduce oxidized lipid molecules and is therefore considered a critical defense mechanism to protect membranes against oxidative stress [29].

A second source of free radical superoxides are NADPH oxidases, multisubunit enzyme complexes located in the cell membrane, which are classified by their catalytic subunit, NOX. Several NOX proteins are expressed in cells of the CNS including neurons, astrocytes, and microglia, with NOX2 as the predominant form in microglia and astrocytes [30, 31]. In healthy cells ROS generation by NADPH oxidases is involved in cell signaling and tissue homeostasis [31, 32]. During infections, activation of NADPH oxidases is strongly increased and the resulting increase in ROS is particularly important as a host defense mechanism. However, excessive NADPH oxidase activation has also been implicated in oxidative stress mediated neurodegeneration [32].

Next to ROS, also reactive nitrogen species (RNS) can contribute to oxidative stress (Figure 1). One example is the generation of the highly reactive peroxynitrite (ONOO^−^) due to interaction of nitric oxide (NO) with superoxide. ONOO^−^ can react with CO_2_ to form the highly reactive radicals ^*∙*^NO_2_ (nitrogen dioxide) and CO_3_^−∙^. NO_2_ and NO can then react further to form N_2_O_3_ (dinitrogen trioxide). NO itself is produced by NO synthase (NOS), which has three isoforms in the CNS, endothelial NOS (eNOS), neuronal NOS (nNOS) identified in neurons, and inducible NOS (iNOS) identified in glial cells [9, 33]. Under physiological conditions, NO is predominantly generated in short bursts by eNOS and nNOS and is, for example, involved in regulation of blood flow, cell differentiation, and neurotransmission [33, 34].

At the cellular level ROS and RNS can cause DNA and protein oxidation as well as lipid peroxidation (Figure 1). The latter is particularly relevant in the CNS due to the high amount of polyunsaturated fatty acids. Therefore oxidative stress can cause damage of cellular membranes and thus compromise cell integrity and viability. Moreover, compared to nuclear DNA the mitochondrial DNA is particularly vulnerable to ROS-mediated damage [23, 35]. Mutations due to oxidative damage may therefore contribute to mitochondrial dysfunction and thus increased ROS production in the ETC [36].

Two main aspects contribute to the vulnerability of the CNS to oxidative stress mediated neurodegeneration: high metabolism and restricted cell renewal. First, the CNS is a metabolically highly active organ, requiring approximately 20% of the total energy consumption of the body. Therefore the CNS contains high amounts of mitochondria, which are particularly active, resulting in high amounts of ROS [37]. Accordingly, since many mitochondrial enzymes require iron for their function, the iron content in CNS cells is particularly high, which promotes the generation of highly reactive ROS species in the mitochondrial matrix [24]. Therefore, mitochondria in the CNS are highly susceptible to ROS-mediated damage resulting in mitochondrial dysfunction. Energy demand is particularly high in neurons to maintain axonal transport and neuronal conduction as well as in oligodendrocytes to maintain myelination. Therefore mitochondrial dysfunction has been associated with axonal degeneration and compromised oligodendrocyte viability [35, 37].

Second, in postmitotic cells such as neurons and oligodendrocytes, nonfunctional proteins and organelles have to be degraded to prevent their cellular accumulation which may eventually cause cell death. Nonfunctional proteins are predominantly degraded in an ubiquitination-dependent manner by the proteasome [38]. Importantly, oxidative stress can promote protein aggregation thereby impairing their degradation by the proteasome [39]. These protein aggregates as well as organelles are mainly degraded by autophagy [40]. The removal of dysfunctional mitochondria by autophagy is especially important, since mitochondrial damage can initiate apoptosis by release of cytochrome C and proapoptotic factors such as SMAC/Diablo [35, 41]. Importantly, in animal models inhibition of autophagy can promote neurodegeneration [42]. The removal of nonfunctional proteins and organelles is especially relevant for neurons due to the highly restricted neurogenesis. Therefore, insufficient clearance is associated with accumulation of damaged molecules and organelles with increasing age [43, 44]. It is therefore not surprising that aging is the major risk factor for the development of neurodegenerative diseases. This is corroborated by the finding that autophagy is reduced in aging CNS cells [45] and that microglia are less efficiently recruited to the areas of neurodegeneration resulting in delayed tissue repair in the aging CNS [46].

### 3.1. Mitochondrial Dysfunction in Alzheimer's Disease

There is strong support for the role of ROS and mitochondrial dysfunction in AD. For example, neurons of AD patients have a high percentage of damaged mitochondria, which may be due to the increased presence of mutations in the mitochondrial DNA [47]. Moreover, AD is characterized by accumulation of iron in the hippocampus, cerebral cortex, and basal nucleus of Meynert, where it colocalizes with AD lesions and may promote oxidative stress [48]. Importantly, oxidative damage of mitochondrial proteins and DNA occurs already in early stages of the disease suggesting a role of oxidative stress in disease progression [47, 49].

As described in Section 2, AD is characterized by the aggregation of A*β* peptide, derived from APP, as well as misfolded tau protein and both have been implicated in mitochondrial dysfunction. In particular, mitochondrial damage may be caused by intracellular APP and/or A*β*, since these proteins can directly bind to the protein import machinery of mitochondria thereby impairing import of mitochondrial proteins [50]. This results in decreased activity of the ETC and consequently in increased ROS production. Moreover, A*β* and tau can deregulate the mitochondrial ETC at distinct sites resulting in mitochondrial dysfunction and oxidative stress [51]. On the other hand, oxidative stress can activate signaling pathways that may affect the processing of APP as well as the phosphorylation of tau. For example, there is evidence that oxidative stress increases the expression of *β*-secretase through activation of c-jun N-terminal kinase (JNK) and p38 MAP kinase thereby promoting the generation of A*β* [52]. Similarly, activation of glycogen synthase kinase 3 increases tau hyperphosphorylation thus promoting formation of neurofibrillary tangles, a hallmark of AD [53, 54].

Interestingly, A*β* can activate NADPH oxidase in primary cultures of cortical neurons thereby causing the generation of ROS within the cells [55]. Furthermore, there is also evidence linking apoE4, the major risk factor to develop sporadic AD, with increased oxidative stress and mitochondrial dysfunction in hippocampal neurons [56].

### 3.2. Mitochondrial Dysfunction in Parkinson's Disease

Dopaminergic neurons are particularly vulnerable to oxidative stress since dopamine metabolism and transport can contribute to ROS production [17, 57, 58]. Indeed, injection of 6-hydroxydopamine (6-OHDA) mimics many of the hallmark characteristics of PD and is widely used as an oxidative stress model for PD [59]. The importance of mitochondrial dysfunction in PD was first implied by the accidental injection of 1-methyl-4-phenyl-1,2,3,6-tetrahydropyridine (MPTP) by drug addicts. MPTP crosses the blood-brain barrier and is taken up by astrocytes where it is metabolized into 1-methyl-4-phenylpyridinuim (MPP^+^). MPP^+^ is a substrate for the dopamine transporter and is taken up selectively into dopaminergic neurons where it inhibits complex I of the mitochondrial electron transport chain ultimately resulting in PD-like symptoms [59, 60]. The relevance of mitochondrial dysfunction in PD is further corroborated by the abundant deletions in mitochondrial DNA in substantia nigra neurons of PD patients [61–63]. Moreover, alterations in the glutathione levels in adult dopaminergic neurons indicate that impaired physiological defense mechanisms against oxidative stress may be involved in the loss of dopaminergic neurons in PD [64, 65].

Importantly, mutations in most genes that have been implicated in hereditary forms of PD, such as *α*-synuclein, parkin, PINK1, and DJ-1, affect mitochondrial function resulting in increased ROS production [66–68]. This results in oxidative damage, mitochondrial dysfunction, and ultimately cell death. Of particular interest is *α*-synuclein, the major component of Lewy bodies, since *α*-synuclein can directly impair the mitochondrial complex I in dopaminergic neurons [69]. Moreover, PD-associated mutations as well as overexpression of *α*-synuclein are implicated in increased ROS production while oxidative stress promotes *α*-synuclein aggregation thus creating a vicious cycle promoting neurodegeneration [70–73].

## 4. Inflammation in Neurodegenerative Disease

**Figure 2:**
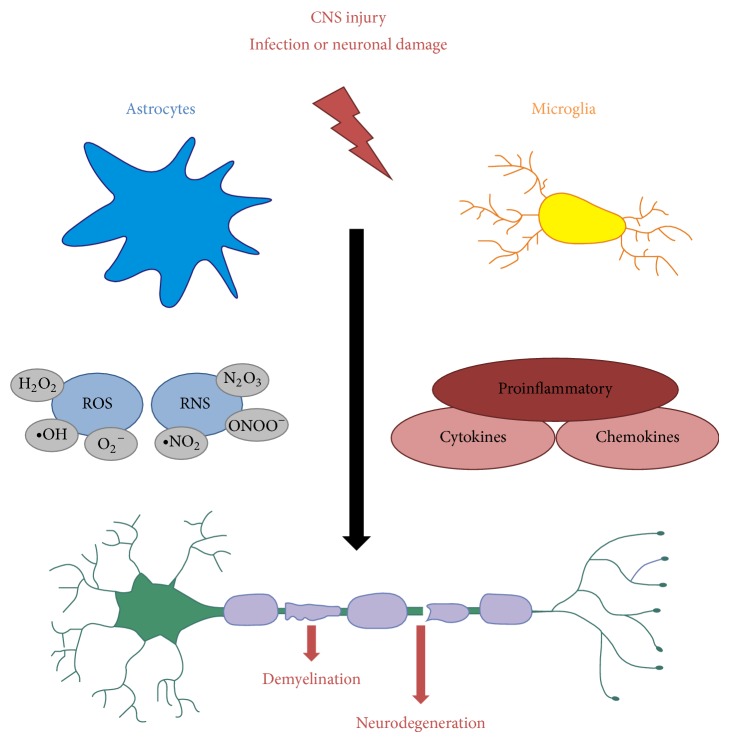
Schematic presentation of the CNS cell mediated demyelination and neurodegeneration. CNS injury, for example, during infection or due to neuronal damage, leads to the activation of astrocytes and microglia. This induces the secretion of ROS, RNS, and proinflammatory cytokines and chemokines. These factors then can promote demyelination and axonal damage, finally leading to neurodegeneration.

So far we have focused on the role of cell intrinsic oxidative stress resulting in neuronal damage. However, during infection or injury, factors are released, the so-called pathogen associated and danger associated molecular patterns (PAMPs and DAMPs), respectively, that are recognized by pattern recognition receptors. Important pattern recognition receptors in the CNS are, among others, the toll-like receptors (TLRs) which are broadly expressed by microglia and astrocytes [2, 74]. Upon TLR-mediated activation both astrocytes and microglia can release cytokines and chemokines as well as ROS, which can either promote neuronal survival or, in case of massive damage as in ischemia or spinal cord injury, may promote inflammation and aggravate neuronal damage [75] (Figure 2). In this context it is relevant to note that there is a strong interdependence of microglia and astrocytes activation. For example, it has been shown that astrogliosis in the substantia nigra leads to local activation of microglia in PD [17, 76]. On the other hand, microglia are required for the induction of iNOS and TNF expression in cultivated astrocytes [77].

In general, inflammation is a protective response to various cell and tissue injuries to destroy and remove the detrimental agents and injured tissues, thereby promoting tissue repair. However, when inflammation is uncontrolled, it can cause excessive cell and tissue damage ultimately leading to destruction of normal tissue and chronic inflammation [10]. This is especially relevant in chronic neurodegenerative diseases such as PD and AD, which usually last over decades. Here, the continuous presence of damaged neurons results in the constant activation of microglia and astrocytes. This generates a neuroinflammatory environment which is thought to promote neurodegeneration [2, 12]. Prolonged activation of astrocytes can also induce the release of extracellular matrix proteins such as chondroitin sulfate that result in the formation of a glial scar. This, on one hand, prevents the spreading of the damaged area but on the other hand restricts tissue regeneration [78, 79]. Moreover, depending on the damage, peripheral macrophages may enter the CNS and under certain conditions, for example, in multiple sclerosis, T and B cells can cross the BBB and enter the CNS parenchyma to mount an adaptive immune response thus further promoting neuroinflammation and degeneration [2, 18].

Important roles of microglia and astrocytes in chronic neurodegeneration have first been suggested due to the increased presence of both cell types around sites of A*β* deposition in AD and in the substantia nigra in PD patients [80–82]. Both A*β* and *α*-synuclein can bind and activate TLRs, in particular TLR2, in the membrane of astrocytes and microglia [2, 17, 83, 84]. On one hand this can promote the phagocytic activity of microglia thus promoting clearance of the protein aggregates from the CNS; on the other hand activation of TLRs can induce the expression of proinflammatory cytokines and chemokines, such as TNF, interleukins (IL), and CCL2 [85], which may contribute to disease progression [2] (Figure 2). Indeed persistent TLR2 activation has been implicated in neuroinflammation and development of neurodegenerative diseases [85], whereas TLR3 may have protective functions [85, 86].

Upon chronic activation microglia and astrocytes can also generate ROS and RNS (Figure 2). Particularly the generation of superoxide by NADPH oxidase has important functions as a host defense mechanism. However, excess superoxide production has also been implicated in neurodegeneration [32]. Moreover, as mentioned in Section 3, superoxide can interact with NO to produce peroxynitrite, which has been shown to be toxic to neuronal cells [87, 88]. Importantly, during inflammation in the CNS the expression of NADPH oxidases and NO synthases, in particular NOX2 and iNOS, is induced in microglia and to some extent also in astrocytes [30, 89]. This results in high superoxide and NO levels resulting in subsequent neuronal damage [32, 33, 90, 91]. Interestingly, oxidative stress in active MS lesions coincides with the increased expression of antioxidative enzymes [92] demonstrating that the CNS responds to increased presence of harmful ROS and RNS by activating respective defense mechanisms (Figure 1). There is, however, evidence that these defense mechanisms are less active in chronic neurodegenerative diseases [64, 93, 94].

Both A*β* and *α*-synuclein can directly activate NOX2 in microglia causing a burst of superoxide [32, 63]. It is therefore not surprising that overactivation of NOX2 due to the continuous presence of A*β* and *α*-synuclein has been implicated in the chronic neurodegeneration in AD and PD patients [95–97]. Similarly, increased expression of iNOS has been detected in astrocytes surrounding A*β*-plaques in AD brains and in microglia in the substantia nigra of PD patients [32, 63]. Importantly, inhibition of iNOS successfully ameliorated neurological symptoms in an animal model of PD strongly supporting the role of NO in chronic neurodegeneration [98].

Particular support for the link of ROS production and inflammation in neurodegeneration is again obtained from the role of mutated *α*-synuclein in PD. As mentioned in Section 3.2, PD-associated mutations in *α*-synuclein amplify its oxidization and aggregation [70, 72]. After release by dying neurons, this oxidized *α*-synuclein subsequently promotes activation of NADPH oxidase in microglia and thus enhances the generation of free ROS thus boosting the oxidization of *α*-synuclein in intact neurons [71, 99–101]. Accordingly, the dual pathogenic mechanism, whereby *α*-synuclein is altered by oxidative stress and the oxidized *α*-synuclein is promoting chronic activation of microglia, creates a feed-forward state for the progressive neuronal death seen in PD [17, 102].

Although oxidative stress is implicated as a causative factor in neurodegenerative disorders, the signaling pathways linking ROS production with neuronal cell death are not well characterized. However, certain ROS-mediated signaling pathways relevant for the induction of inflammatory responses have been elucidated [10]. The activation of signaling pathways by ROS requires recognition of environmental changes in the redox state. Under physiological conditions this leads to a temporary activation of signaling pathways. However, abnormally large concentrations of ROS/RNS may lead to permanent changes in signal transduction and gene expression, typical for disease states. Main signaling mechanisms that are regulated by redox signaling are activation of the transcription factors AP-1, HIF-1, and NF*κ*B as well as induction of the stress-responsive protein kinases JNK and p38 MAP kinase [103, 104]. NF*κ*B, in particular, has been recognized as a major player in governing cellular responses to oxidative stress.

The family of NF*κ*B consists of five members that are characterized by a Rel homology domain, which is required for dimerization and DNA binding. RelA (p65), RelB, and cRel contain a transactivation domain required to activate transcription of NF*κ*B target genes. The other two members, p100 and p105, are formed as precursor proteins, which are processed by the proteasome to p52 and p50, respectively. Although the NF*κ*B family members can form several homo- and heterodimers, two main signaling pathways have been described, the canonical and the noncanonical NF*κ*B pathways, which are usually mediated by p65/p50 and RelB/p52 dimers, respectively. NF*κ*B-activity is inhibited by I*κ*B proteins which prevent translocation of NF*κ*B into the nucleus [105–107]. NF*κ*B activation can have various physiological consequences and has been implicated in cell proliferation, differentiation, and survival by the induction of antiapoptotic proteins. However, NF*κ*B is best known for its role in the immune system where it acts as an important positive regulator of the inflammatory response, predominantly, but not exclusively, by activation of the canonical NF*κ*B signaling pathway [105]. Of particular relevance is that activation of TLRs by binding to PAMPS or DAMPS and thus also the binding of A*β* and *α*-synuclein to TLR2 results in the activation of the canonical NF*κ*B-signaling pathway [108]. Similarly, the canonical NF*κ*B pathway is predominantly activated by various proinflammatory cytokines such as TNF and IL-1 [109, 110] and, at least in immune cells, also by ROS [110].

As mentioned above the physiological consequence of NF*κ*B activation can be highly diverse. Of relevance for the development of chronic inflammatory and neurodegenerative diseases is the NF*κ*B-mediated induction of inflammatory mediators, in particular cytokines such as IL-6, IL-8, and TNF, and adhesion molecules thus promoting recruitment of immune cells to inflamed tissues [105, 107]. Importantly, NF*κ*B is a potent inducer of NOX2 and iNOS and thus contributes to the generation of ROS/RNS under proinflammatory conditions [107]. Moreover, NF*κ*B can induce the expression of COX-2 and cPLA2, which are involved in the generation of prostaglandins thus further promoting recruitment of inflammatory cells [10, 111]. Since superoxide is produced during the generation of prostaglandins, this may also contribute to oxidative stress [107]. This role of NF*κ*B demonstrates the strong interrelationship between ROS/RNS production and the induction of proinflammatory cytokines, which results in enhanced cell damage and thus aggravates neurodegeneration (see Figure 1).

In contrast to NF*κ*B, which, in addition to its proinflammatory functions, promotes usually cell survival, sustained activation of p38 or JNK by ROS can promote apoptosis, for example, by cleaving the antiapoptotic protein Bid, thereby destabilizing the mitochondrial membrane [112, 113]. JNK is of particular relevance for the development of AD, since sustained activation of this kinase can result in hyperphosphorylation of tau protein and is involved in A*β* oligomerization as well as synaptic dysfunction and cognitive decline in animal models of AD [114–116]. Several JNK isoforms exist and* in vitro* as well as* in vivo* animal studies have revealed that JNK3 is particularly relevant for AD and PD pathology, although for PD an additional role of JNK2 has been suggested [117]. Moreover, JNK3 levels are elevated in postmortem brains of AD patients, further supporting the role of JNK3 in chronic neurodegenerative diseases [117].

## 5. Tumor Necrosis Factor

As mentioned in the previous section microglia and astrocytes release a battery of cytokines upon activation. Accordingly, due to constant activation of these cells in chronic neurodegenerative diseases the affected areas are characterized by an increased amount of proinflammatory cytokines such as interleukin-1, interleukin-6, and tumor necrosis factor (TNF) [2, 118]. TNF, in particular, has been implicated as an important factor for the onset and perpetuation of neurodegenerative diseases, since increased levels of this cytokine are present in the affected areas in many neurodegenerative diseases [119–121].

TNF is synthesized as a type II transmembrane protein that self-assembles into noncovalently linked homotrimers. Proteolytic cleavage of the ectodomain of this initial membrane-bound form of TNF (memTNF) by the TNF-*α* converting enzyme (TACE) results in the release of soluble TNF (sTNF). TNF can bind to two membrane receptors, TNF receptor (TNFR) 1 and TNFR2. Whereas TNFR1 is constitutively expressed on virtually all tissue cells, the expression pattern of TNFR2 is restricted to cells of the immune systems, especially regulatory T cells, endothelial, and neuronal tissues and its expression can be highly regulated by the cellular activation status. Both receptors are typical type I transmembrane proteins with extracellular and intracellular domains of about equal sizes and single transmembrane domains [109].

The extracellular domains of both receptors are quite similar comprising four cysteine rich domains, which are required for ligand binding but also carry a homophilic interaction motif. Interestingly, whereas TNFR1 can be equally well activated by both memTNF and sTNF, the activation of TNFR2 is dependent on the presence of memTNF [122]. A further level of complexity is added by the shedding of the TNFR ectodomains, for example, by TACE, which can be increased during inflammation [123]. Shed receptor ectodomains bind to TNF and thus can act as TNF antagonists indicating attempts to regulate the inflammatory response. This concept is, for example, supported by the high levels of the anti-inflammatory cytokine interleukin-10 in active MS-lesions [124] (see also Figure 1).

**Figure 3:**
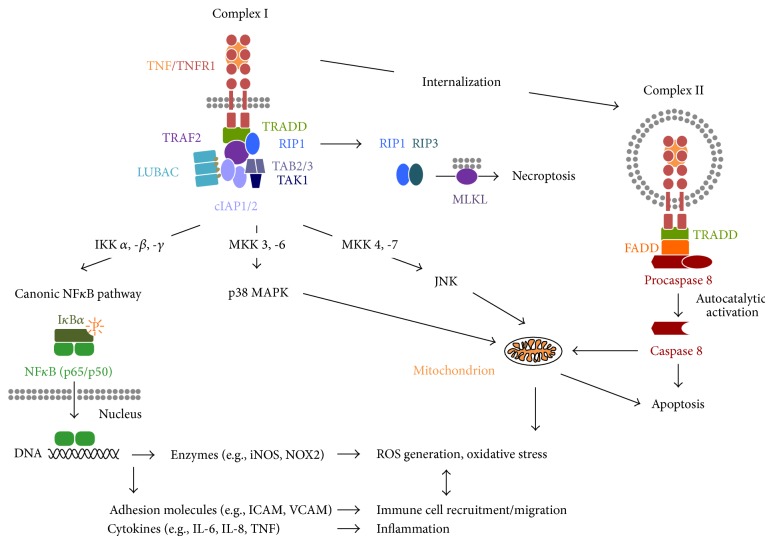
Schematic illustration of the major TNFR1-mediated signaling pathways. After binding of TNF to TNFR1 the signaling complex I is formed in the plasma membrane consisting of TRADD, RIP1, TRAF2, and cIAP1/2 and the protein complexes TAB2/TAB3/TAK1 and LUBAC. This complex I mediates the activation of the NF*κ*B pathway via activation of the IKK complex resulting in phosphorylation and degradation of I*κ*B and translocation of NF*κ*B to the nucleus. NF*κ*B binding to the DNA can then, for example, induce expression of proinflammatory cytokines, cell adhesion molecules, and ROS generating enzymes. Moreover, complex I can induce, via distinct MAP kinase kinases (MKK), the activation of the stress-activated kinases p38 MAPK and JNK, which can both induce gene transcription (not shown). In the cytosol, sustained activation of p38 and JNK can induce cell death via apoptosis. After TNFR1 internalization, a distinct signaling complex II is formed consisting of TRADD, FADD, and the procaspase 8. After autoproteolytic activation caspase 8 can cleave RIP1 and RIP3, which is followed by the induction of apoptosis either directly or by targeting the mitochondrion. If caspase 8 activity is insufficient, RIP1 and RIP3 can induce the alternative cell death program of necroptosis via their kinase activities. Here, a central step is the phosphorylation of MLKL by RIP3.

The structure of the intracellular signaling domains of both receptors is highly distinct and defines them as representatives of the two main subgroups of the TNFR family, the death domain- (DD-) containing receptors (TNFR1) and the TRAF-interacting receptors (TNFR2), respectively [109]. The DD of TNFR1 can upon TNF binding interact with other DD-containing proteins, such as TRADD (TNF receptor 1 associated protein with death domain). TRADD functions as an assembly platform and recruits the TNF receptor-associated factor 2 (TRAF2), cellular inhibitor of apoptosis proteins (cIAPs) 1 and 2, and the receptor interacting protein kinase 1 (RIP1). The cIAPs are E3 ubiquitin ligases and polyubiquitinate themselves, TRAF2 and RIP1. This creates docking platforms for the linear ubiquitin assembly complex (LUBAC) as well as the transforming growth factor-*β*-activating kinase1 (TAK1) and its associated proteins TAK1-binding protein2 (TAB2) and TAB3 (Figure 3). This membrane-associated primary TNFR1 signaling complex I can induce several signaling pathways, with the canonical NF*κ*B signaling pathway being the most common and best characterized. Here, LUBAC modifies NEMO, the regulatory subunit of the I*κ*B kinase (IKK) complex, by linear ubiquitination, while IKK*β*, one of the catalytic subunits of the IKK-complex, associates with ubiquitinated RIP1 resulting ultimately in activation of the complete IKK-complex [125]. Subsequently, IKK phosphorylates I*κ*B proteins resulting in their ubiquitination and proteasomal degradation, which enables nuclear translocation and DNA binding of NF*κ*B dimers (Figure 3). As mentioned in the previous chapter, NF*κ*B in turn can induce the transcription of many genes which promote inflammation and oxidative stress. These include cell adhesion molecules, for example, ICAM and VCAM, ROS-producing enzymes, for example, iNOS and NOX2, as well as cytokines, for example, IL-6, IL-8, and, importantly, TNF [106, 107, 126] thus potentially resulting in an amplification loop of TNF signaling (Figure 3). Next to the NF*κ*B pathway, complex I can initiate, by binding and activation of distinct MAP kinase kinases (MKK), the activation of p38 MAP kinase and JNK, two important pathways which are associated with the induction of apoptosis via mitochondrial dysfunction (Figure 3) and the development of neurodegenerative diseases [104, 127, 128].

After complex I internalization TRAF2 and cIAPs dissociate from TNFR1 and a secondary proapoptotic signaling complex II is formed by recruitment of the adaptor protein Fas-associated death domain protein (FADD) and the procaspase 8 to the receptor complex resulting in the formation of the death inducing signaling complex (DISC) [129, 130]. After its deubiquitination, RIP1 can also bind to this complex via FADD. However, autoproteolytically activated caspase 8 degrades RIP1 ultimately resulting in the induction of apoptosis [131]. Moreover, TNFR1 signaling complex II can target the mitochondrion, for example, by induction of mitochondrial ROS formation, thus further promoting apoptosis [132, 133]. Beside induction of classical apoptosis via complex II, TNFR1 is also capable of initiating cell death by a form of controlled necrosis called necroptosis [134]. Necroptosis is activated especially under conditions of inhibited apoptosis by the kinase activities of RIP1 and RIP3, resulting in the phosphorylation of mixed lineage kinase domain-like protein (MLKL). Although it was initially suggested that RIP3-mediated phosphorylation of MLKL causes necroptosis due to mitochondrial dysfunction, recent evidence shows that translocation of MLKL to the plasma membrane is required for TNF-induced necroptosis [135, 136].

In contrast to TNFR1, TNFR2 does not contain a DD and therefore cannot directly induce cell death. However, TNFR2 can directly interact with TRAF-proteins, namely, TRAF 1, 2, and 3, as well as cIAPs and can thus influence TNFR1 signaling [137–139]. Signaling pathways initiated by TNFR2 activation include the JNK pathway [138] as well as both the canonical [140] and the noncanonical [141] NF*κ*B signaling pathways. Moreover, by an as yet not fully elucidated mechanism TNFR2 can activate the phosphatidyl inositol (PI) 3-kinase/Akt pathway thus promoting cell survival and proliferation [142–144]. In contrast to TNFR1 the composition of the protein complex(es) mediating TNFR2-signaling has not yet been identified, although very recently mitochondrial aminopeptidase P3 (mAPP3) has been identified as member of the TNFR2 signaling complex involved in JNK-activation [145].

## 6. General Role of TNF in Inflammation

TNF is the prototypic member of a large family of cytokines that play an important role in the regulation of the innate and adaptive immune system [146]. TNF itself is a key player in the initiation and orchestration of inflammation and immunity [109]. Generally, TNF is known as a powerful proinflammatory molecule with stimulatory activities for most cells of the immune system. Monocytes and macrophages are the major sources of TNF synthesis* in vivo*, although many other cell types are also capable of producing TNF under certain circumstances. TNF acts as a costimulator for natural killer cells, activated B and T lymphocytes, and it enhances the pathogen-directed cytotoxicity of monocytes, neutrophils, and eosinophils.

Of great importance are the stimulatory effects on endothelial cells resulting in enhanced surface expression of adhesion molecules, a central step for the recruitment of immune cells, for example, neutrophils, lymphocytes, and monocytes, which in the case of the CNS might be followed by transendothelial cell migration into neuronal tissues [147, 148]. This is a critical step for the development of inflammatory diseases of the CNS and therapeutics targeting the transmigration of immune cells via the BBB are already used in the clinic, such as natalizumab for the treatment of MS [149, 150].

The pleiotropic functions of TNF in regulation of the adaptive and innate immune response explain why deregulated TNF production due to overreaction of the host or expression in an inappropriate location can lead to major pathogenic consequences. Indeed, persistently elevated levels of TNF have been implicated in chronic inflammation and have been associated with a variety of diseases including autoimmune diseases and neurodegenerative diseases. Blocking of TNF signaling has therefore been evaluated in various inflammatory diseases and is successfully used for treatment of autoimmune diseases such as rheumatoid arthritis, Crohn's disease, and psoriasis [14, 151, 152].

Interestingly, increased serum TNF levels due to systemic inflammation have been associated with an increased cognitive decline in AD and PD suggesting a link between systemic inflammation and neurodegeneration [5, 153]. Since MS is predominantly driven by a systemic immune response, anti-TNF therapies for MS had been initiated. However, despite promising results in mouse models of MS, clinical trials with TNF neutralizing reagents in MS patients failed to ameliorate the disease and in some cases even led to disease exacerbation [154]. Although the cause for the failure of anti-TNF treatment in MS was not clear at that time, it is by now apparent that the distinct roles of TNFR1 and TNFR2 in immune regulation and tissue regeneration provide a mechanistic explanation. Thus, TNF has a dual role in neurodegenerative diseases. Whereas TNFR1 is predominantly associated with inflammation and neurodegeneration, TNFR2 is involved in tissue regeneration and neuroprotection [14]. In the following we will address these distinct functions of the two TNFRs in more detail focusing in particular on their role in oxidative stress mediated neurodegeneration.

## 7. Role of TNF in Neurodegenerative Diseases

Although other members of the TNF superfamily, for example, Fas ligand and TRAIL, have been linked to neuroinflammation [155], TNF clearly has a predominant role in the context of chronic neurodegenerative diseases. In the following we will therefore restrict our discussion on TNF. In acute or chronic neurodegenerative disease, TNF is released predominantly by activated microglia and may contribute to primary or secondary tissue injury [14]. Indeed, injection of TNF into the median forebrain bundle of rats can directly cause the degradation of dopaminergic neurons in the substantia nigra [156]. A detrimental effect of TNF in the CNS has also been shown by continuous CNS-selective TNF expression in transgenic mice. This causes spontaneous chronic inflammation and leads to early death [157]. In contrast, a moderate neuron-selective TNF expression in the CNS under control of the NMDA receptor subunit 2b (NR2b) promotor does neither cause inflammation nor affects normal CNS development and function. Rather, NR2b-TNF transgenic neurons are protected from excitotoxic cell death induced by NMDA receptor agonists, indicating a direct neuroprotective role of memTNF [142].

Interestingly, distinct effects of TNF in the CNS have also been revealed by using TNFR knockout mice in MPTP-induced neurotoxicity. In this model, deficiency for both TNFRs protects against MPTP-induced striatal dopaminergic neurotoxicity, suggesting a role for TNF in neurodegeneration in PD. In the hippocampus, however, TNFR-deficient mice showed exacerbated neuronal damage after MPTP treatment. Taken together, the results are indicative of a region-specific and dual role for TNF in the brain: a promoter of neurodegeneration in the striatum and a protector against neurodegeneration in the hippocampus [158].

A direct role of TNF in PD is also suggested by genetic studies, which identified polymorphisms in the TNF gene, which are associated with an increased risk to develop sporadic PD. In contrast the role of genetic variations of TNF is less clear for the development of AD [14]. Importantly, however, perispinal application of the TNF-inhibitor etanercept significantly improved symptoms of AD patients in an open-label pilot study, providing strong evidence for a role of TNF in AD [159].

### 7.1. Role of TNF in Neurodegeneration

There is increasing evidence that the detrimental effects of TNF in chronic neurodegenerative diseases are largely mediated by sTNF. For example, transgenic mice expressing a mutant TNF that cannot be cleaved, thus preventing the formation of sTNF, are largely protected against the induction of autoimmune diseases [160, 161]. Since sTNF predominantly activates TNFR1 this receptor has been implicated in autoimmune diseases and neurodegeneration. Indeed, mutations in TNFR1 are responsible for the development of TNF receptor-associated periodic syndrome (TRAPS), an autoinflammatory disorder characterized by periodic fever episodes. Interestingly, mitochondrial ROS are elevated in TRAPS patients and promote production of proinflammatory cytokines [162].

Importantly, polymorphisms in the TNFR1 gene have been associated with the risk to develop PD or MS [14, 163, 164]. Moreover, the use of TNFR knockout mice revealed that TNFR1 promotes neurodegeneration in retinal ischemia [165], is important for the development of pain-induced depressive behavior [166], and is essential for the development of MOG^35-55^ induced EAE [167–169]. Indeed, inhibition of TNFR1 by a specific antibody strongly ameliorates development of MOG^35-55^ induced EAE [169] and similar results were obtained when a dominant negative TNF (dnTNF) was used that forms heterotrimers with wild type sTNF and in consequence prevents binding of sTNF to TNFR1 [170, 171]. Furthermore, dnTNF also protects against loss of dopaminergic neurons in animal models of PD and AD [172–174]. The evidence supporting that signaling of sTNF via TNFR1 promotes neurodegeneration is summarized in Table 1.

TNFR1 can exacerbate neurodegeneration at several routes, which are mainly mediated by activation of NF*κ*B. First, as mentioned in Section 4, activation of the NF*κ*B signaling pathway in endothelial cells can induce the expression of cell adhesion molecules thereby promoting the transmigration of immune cells into the CNS parenchyma [180]. Moreover, in glial cells, in particular microglia and astrocytes, TNF-mediated activation of the NF*κ*B signaling pathway can induce the production of proinflammatory cytokines such as IL-6, IL-8, and TNF itself. Enhanced TNFR1 signaling will thus greatly enhance the inflammatory response thereby promoting neurodegeneration. Indeed, astrocyte-specific inhibition of NF*κ*B signaling in transgenic mice strongly suppresses the inflammatory response of the CNS in various animal disease models, such as spinal cord injury, EAE, and optic neuritis, thereby reducing neurodegeneration and improving functional recovery [181–183].

Next to the induction of proinflammatory signaling pathways, TNF can contribute to chronic neurodegeneration by promoting the generation and release of ROS and RNS. Indeed, the high systemic toxicity of TNF has been related to excessive induction of NO resulting in vasodilatory shock [184]. In the CNS TNF can, together with interferon gamma (IFN*γ*), increase the expression of iNOS in microglia leading to the secretion of NO [185]. Moreover, in rat spinal cord explants TNF activation induced expression of iNOS resulting in protein oxidation and nitration, thereby increasing the percentage of motor neurons immunoreactive for nitrotyrosine and finally causing the death of the neurons [186]. In this model TNF induced iNOS in an NF*κ*B-dependent manner again demonstrating the relevance of this signaling pathway for the role of TNF in neurodegeneration. Of relevance for AD, the intracerebroventricular injection of A*β* in mice caused marked deficits of learning and memory, which were greatly reduced by TNF and iNOS inhibitors. Importantly, a similar reduction of AD symptoms was observed in TNFR1-deficient mice demonstrating the role of TNFR1 in this animal model of AD [187].

Moreover, TNF can also directly exacerbate the formation of ROS by activating NADPH oxidases thus inducing the production of superoxide [31]. For example, TNF can induce the expression of distinct NOX subunits in different cells [188] and can promote NOX2 activity in an NF*κ*B-dependent manner [189]. Importantly, TNF stimulates ROS production by isolated microglia through the NADPH oxidase system [190]. A specific role of TNFR1 in the activation of NADPH oxidases has been suggested by the finding that riboflavin kinase can induce the association of TNFR1 with Nox1 and Nox2 via the common NADPH oxidase subunit p22(phox) [191].

Another mechanism by which TNF, via TNFR1, can promote the generation of ROS is the activation of COX-2, another NF*κ*B target gene. Indeed, it has been shown that TNFR1 is required for the efficient induction of COX-2 in activated macrophages [192]. Importantly, in an animal model of AD, TNF mediates, likely via TNFR1, the A*β*-induced activation of COX-2, which is correlated to the cognitive decline of the animals [193].

Although sustained activation of JNK, in particular JNK3, has been implicated in neurodegeneration, the evidence for a role of TNF-mediated JNK-activation in chronic neurodegeneration is scarce. One example is the role of TNFR1-induced JNK-activation in the death of retinal ganglion cells following optic nerve crush injury [194]. Of relevance for the toxic effect of TNF in PD is the finding that TNF-induced activation of the MAP kinase pathway can activate JNK which in turn can promote death of dopaminergic neurons [195].

Next to the indirect neurodegenerative effects of TNF by promoting neuroinflammation and oxidative stress mediated by glial cells,* in vitro *studies have shown that TNF, via TNFR1, can also mediate direct apoptosis of neurons by activation of caspase 8 [175]. Elevated levels of TNF and activated caspase 8 in spinal cord injury models further support the notion of TNF mediated direct neuronal cell death* in vivo* [196]. Whether TNFR1-induced necroptosis, which may promote an inflammatory response due to the release of cytoplasmic molecules that can act as DAMPs, plays a significant role in chronic neurodegenerative diseases is currently unclear [163].

### 7.2. Role of TNF in Neuroregeneration

Whereas the proinflammatory and neurodegenerative effects of TNF are primarily mediated by sTNF and thus by TNFR1, signaling via memTNF, predominantly via TNFR2, is mainly neuroprotective and supports tissue homeostasis and regeneration. The relevance of TNFR2 signaling for neuroprotection and tissue regeneration had initially been shown in TNFR2 knockout mice. Here, a role for TNFR2 in tissue regeneration has been described in the cuprizone model of reversible demyelination. In this model proliferation of oligodendrocyte progenitor cells (OPCs) and remyelination are significantly delayed in TNF and TNFR2 knockout mice, demonstrating that tissue regeneration is dependent on the signaling of TNF via TNFR2 [176] (Table 1). Moreover, TNFR2 knockout mice show significantly exacerbated neurodegeneration in EAE and retinal ischemia [165, 167, 168]. Finally, TNF can protect primary cortical neurons from TNFR1 knockout mice against glutamate-induced excitotoxicity, whereas neurons from TNFR2 knockout mice are not protected [142]. Interestingly, this protective effect of TNFR2 was dependent on NF*κ*B activation. Indeed, whereas induction of NF*κ*B in astrocytes and microglia promotes inflammation and neurodegeneration (see Section 7.1), neuronal NFkB appears to be neuroprotective [197] and activation of NF*κ*B by memTNF can be neuroprotective in EAE [171]. In this context it is of interest that TNFR2 stimulation can induce a long-lasting nuclear translocation of NF*κ*B in cortical neurons, which has been related to its protective effect against excitotoxicity [142]. This persistent NF*κ*B activation may be facilitated by the predominant activation of TNFR2 by memTNF. The direct cell-cell contact in tightly packed neuronal tissue allows stable ligand-receptor interactions, favoring TNFR2 activation.

Besides the NF*κ*B pathway, also the PI 3-kinase/Akt survival pathway has been implicated in TNFR2 mediated neuroprotection [142, 143]. More recently, soluble TNFR2 specific agonists have been generated, which mimic the activity of membrane TNF and selectively activate TNFR2 [144], thereby providing a tool to dissect the mechanisms that are involved in TNFR2 mediated neuroprotection.* In vitro* studies have revealed that neuroprotective effects of TNFR2 indeed require the activation of the PI 3-kinase/Akt pathway [144, 177]. Importantly, dopaminergic neurons were protected from H_2_O_2_ or 6-OHDA induced cell death by selective activation of TNFR2 after the toxic insult [144]. Although the exact molecular mechanisms of this TNFR2-PI 3 kinase/Akt-mediated neuroprotection are still unresolved, the role of Akt and its downstream targets are well known to promote cell survival through interference with cell death pathways by inactivating components of the apoptotic machinery or activation of antiapoptotic proteins [198–200]. Next to these indirect effects, Akt can directly block cell death after mitochondrial cytochrome C release, most likely by phosphorylating caspase 9 at serine 196 [201], thereby inactivating the caspase.

On a mechanistic level, TNFR2 activation promotes the release of anti-inflammatory and neurotrophic factors from astrocytes and microglia [177, 178, 202], which may explain some of the protective and regenerative effects of TNFR2. In particular, astrocyte-derived factors, namely, CXCL12 and leukemia inhibitory factor (LIF), promote oligodendrocyte differentiation and may thus support remyelination [177, 178]. Moreover, TNFR2 activation in OPCs enhances the expression of antiapoptotic and antioxidative proteins such as BCL-2 and SOD2 which may stabilize the mitochondrial membrane [203, 204] and thus might contribute to the observed TNFR2-mediated protection of OPCs against H_2_O_2_-induced cell death [179]. These results support the concept that specific TNFR2 activators might present a novel therapeutic concept in neurodegenerative diseases [205, 206].

## 8. Conclusion

The two most common features of neurodegenerative diseases are sustained oxidative stress and inflammation. Here, we have provided an overview on the interrelation between these two hallmarks of neurodegeneration. Of particular importance is the excessive generation of ROS, for example, due to mitochondrial dysfunction, which causes neuronal damage and thus the release of cytosolic factors that activate neighboring microglia and astrocytes. These cells respond by the release of proinflammatory cytokines as well as ROS and RNS thus further promoting the inflammatory response and exacerbating the neuronal damage. Accordingly, persistent activation of glia cells can ultimately result in an amplification loop resulting in chronic neurodegeneration.

**Figure 4:**
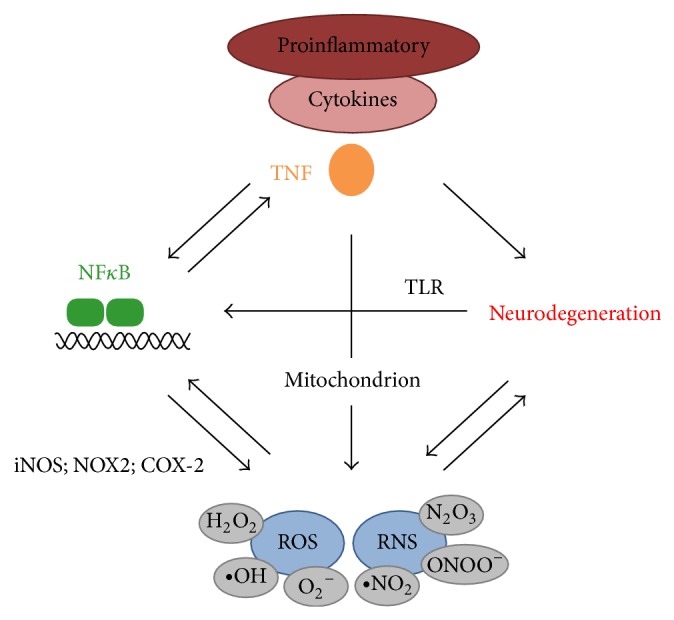
Interrelation of ROS and inflammatory cytokines in neurodegeneration. An initial neuronal damage can promote inflammation by activating the canonical NF*κ*B pathway in glia cells via TLR activation resulting in the expression of proinflammatory cytokines and generation of ROS/RNS. Proinflammatory cytokines, in particular TNF, can promote neurodegeneration by further activating NF*κ*B in glia cells, but also by damaging mitochondria in neurons resulting in increased ROS formation or by directly inducing neuronal cell death. ROS can further activate NF*κ*B signaling in glia cells thereby promoting a sustained proinflammatory response. In addition, excess ROS/RNS formation by mitochondrial damage or by activated microglia and astrocytes can exacerbate neurodegeneration by damaging DNA, proteins, and membranes. Moreover, altered neuronal proteins, such as A*β* and *α*-synuclein, can promote ROS formation, for example, by impairing mitochondrial function. This interrelation of ROS promoting inflammation and TNF promoting ROS production can, when uncontrolled, ultimately result in chronic neurodegeneration.

TNF is a key cytokine of the immune system that initiates and promotes inflammation, which under uncontrolled conditions may lead to the development of neurodegenerative diseases. The activation of the NF*κ*B pathway in glia cells is a key mediator of these detrimental effects of TNF leading on one hand to the elevated production of proinflammatory cytokines and on the other hand to the production of iNOS, COX-2, and NOX subunits, thereby activating NADPH oxidases, ultimately leading to the production of ROS. TNF itself is a response gene of the NF*κ*B pathway and, moreover, ROS can activate the NF*κ*B pathway thereby amplifying the TNF/ROS/NF*κ*B responses. In consequence, this aggravates the neuronal damage, which further promotes neuroinflammation ultimately resulting in a feed-forward loop that causes chronic neurodegeneration. This central role of NF*κ*B and the resulting strong cross talk between proinflammatory cytokines, mainly TNF, and ROS/RNS emphasize the interrelation between inflammation and oxidative stress in neurodegeneration (Figure 4).

Translating these findings into the production of novel therapies to attenuate neurodegeneration is, however, challenging due to the distinct effects of TNF via its two receptors, which is exemplified by the failure of TNF-inhibitors in MS treatment [154]. Since the detrimental effects of TNF are predominantly mediated by sTNF via TNFR1, inhibitors targeting specifically sTNF and/or TNFR1 are required. Importantly, the generation of such specific inhibitors has gained momentum in recent years and some of them have already been successfully used in animal models of neurodegenerative diseases [170–174, 180]. They show therefore promise for the treatment of human chronic neurodegenerative diseases.

## Figures and Tables

**Table 1:** Role of TNF signaling in neurodegenerative diseases.

sTNF/TNFR1 signaling	
sTNF is necessary for the induction of autoimmune diseases [160]	
Association of TNF and TNFR1 polymorphisms with PD risk [14, 163]	
Association of TNFR1 polymorphism with MS risk [164]	
TNFR1 promotes neurodegeneration in retinal ischemia [165]	
TNFR1 mediates development of pain-induced depressive behavior [166]	
TNFR1 is essential for the development of MOG^35–55^ induced EAE [167–169]	
Inhibition of TNFR1 signaling strongly ameliorates EAE development [169]	
Blocking of sTNF improves functional outcome in EAE [170, 171]	
Blocking sTNF attenuates neurodegeneration in AD and PD models [172–174]	
TNFR1 mediates direct apoptosis of neurons via caspase 8 [175]	

memTNF/TNFR2 signaling	

TNFR2^−/−^ mice show exacerbated neurodegeneration in EAE model [167–169]	
TNFR2^−/−^ mice show exacerbated neurodegeneration in retinal ischemia [165]	
TNFR2 promotes OPC proliferation and remyelination [176]	
TNFR2 protects neurons against glutamate-induced excitotoxicity [142, 143]	
memTNF dependent activation of NFkB is neuroprotective in EAE [171]	
TNFR2 rescues neurons from oxidative stress-induced cell death [144]	
TNFR2 promotes oligodendrocyte maturation [177]	
TNFR2 promotes oligodendrocyte proliferation and maturation [178]	
TNFR2 protects OPCs against oxidative stress [179]	
